# Improving access to safe abortion in a rural primary care setting in India: experience of a service delivery intervention

**DOI:** 10.1186/s12978-016-0157-5

**Published:** 2016-05-10

**Authors:** Kirti Iyengar, Sharad D. Iyengar

**Affiliations:** Action Research & Training for Health, Satyam, Ramgiri, Badgaon, Udaipur, Rajasthan 313011 India; Department of Women’s & Children’s Health, Karolinska Institutet, Solna Stockholm, 17176 Sweden

**Keywords:** Medical abortion, Mifepristone, Primary care settings, India, Safe abortion

## Abstract

**Background:**

Abortion services were legalized in India in 1972, however, the access to safe abortion services is restricted, especially in rural areas. In 2002, medical abortion using mifepristone- misoprostol was approved for termination of pregnancy, however, its use has been limited in primary care settings.

**Methods:**

This paper describes a service delivery intervention for women attending with unwanted pregnancies over 14 years in four primary care clinics of Rajasthan, India. Prospective data was collected to document the profile of women, method of abortion provided, contraceptive use and follow-up rates after abortion. This analysis includes data collected during August 2001-March 2015.

**Results:**

A total of 9076 women with unwanted pregnancies sought care from these clinics, and abortion services were provided to 70 % of these. Most abortion seekers were married, had one or more children. After 2003, the use of medical abortion increased over the years and ultimately accounted for 99 % of all abortions in 2014. About half the women returned for a follow-up visit, while the proportion using contraceptives declined from 74 % to 52 % from 2001 to 2014.

**Conclusions:**

The results of our intervention indicate that integrating medical abortion into primary care settings is feasible and has a potential to improve access to safe abortion services in rural areas. Our experience can be used to guide program managers and service providers about reducing barriers and making abortion services more accessible to women.

## Background

Abortion services were legalized in India in 1972 through the Medical Termination of Pregnancy Act (MTP Act), which permitted provision of abortion services under specified conditions up to 20 weeks of pregnancyn [1]. The law allows abortion to be provided from public and certified private facilities, and only by gynecologists or certified doctors who have received special training. However, access to safe abortion services continues to be poor [2], especially in rural areas of the country, due to skewed distribution of abortion facilities and providers, barriers related to service provision. For example, a study of licensed facilities across all 33 districts of Rajasthan in 2009-10 revealed that there were 0.85 certified abortion facilities per 100,000 population in rural blocks as compared to 3.65 in urban blocks [3]. In 2002, medical abortion using mifepristone and misoprostol was approved for termination of pregnancy up to 49 days of gestation [4], and in 2006, the Drug Controller of India appproved use of a combipack of the same drugs up to 9 weeks of gestation [5]. Medical abortion drugs however remain unavailable in most primary care facilities. Since provision of medical abortion does not require the provider to have skills for surgical evacuation, it could be offered from primary care facilities that lack staff skilled in performing vacuum aspiration.

Even in facilities that provide abortion services, various other barriers prevent women from accessing safe abortion. These include lack of confidentiality, and insistence on husband’s or relative’s consent, even though not mandated by the law. Several public health facilities are known to provide abortion services only on the condition that women adopt either sterilization or copper IUD [6] after the procedure. Meanwhile, the cost of abortion services in private facilities is unaffordable for many women [7]. Lastly, some facilities do not offer women a choice of abortion method, and several others prefer to use dilation and curettage [2] with its attendant risks and admission requirements, hence women seeking medical abortion pills tend to avoid such facilities.

Women in Rajasthan endure social disadvantages that make it difficult for them to seek services from a distant urban facility-only 48 % women are literate [8], and 62 % married before the age of 18 years. They lack autonomy in terms of mobility- data from 2005-06 reveals that only 32 % of women were allowed to go alone to the market, the health facility, and places outside the village/community [9]. The use of reversible contraceptive methods is low- 4.6 % couples used a modern method of contraception before the first child, and 16.5 % did so between their first and second child [9]. Although women shoulder a heavy work burden, most of their work is unpaid farming or domestic work. The maternal mortality ratio of the state is higher than the average for the country, at 254 per 100,000 live births, of these, 10 % deaths are due to unsafe abortion [10].

Action Research and Training for Health (ARTH), an Indian non-profit public health organization, implements a rural field service program that includes community outreach in 50 interior villages with a population of about 70,000, nearly half of which belongs to an under-privileged tribal community. Three rural health centers located within this area, provide a range of primary care services including antenatal care, 24-h delivery and newborn care, first trimester abortions and reversible methods of contraception. First trimester abortion services are provided using manual vacuum aspiration (MVA) and medical abortion (MA). The MVA services have been available since 1999 [11], while medical abortion was introduced in 2003.

The aim of the present analysis is to document trends and profile of abortion seekers and patterns of care seeking in a rural service in a low resource setting of Rajasthan, India, and to inform the programs on how medical abortion could be integrated into abortion services services.

## Methods

The present analysis is based on data on women coming with unwanted pregnancies to any one of three primary care centres (located between 25 and 55 km from Udaipur city) operated by ARTH, during the period Aug 2001 to March 2015. These facilities provide a range of reproductive and child health services through a team comprising day-visiting gynecologists and locally resident nurse-midwives. Data was analysed to document the profile of women, method of abortion provided, contraceptive use and follow-up rates after abortion. These facilities started providing abortion services at different times, in 1999, 2003 and 2011.

At all health centres, visiting gynecologists provided first trimester abortion services on one or two fixed days per week, while trained nurse midwives assisted with counseling, screening for eligibility, provision of services and post-abortion care. Assessment of gestational age was done using bimanual examination. Ultrasound was not used at any of the clinics. If a sonography was indicated, women were referred to a facility in Udaipur city. Women who were above 12 weeks of gestational age or had a medical condition that required higher level of care or additional investigations were referred to facilities in Udaipur city for abortion. Up to 2002, only manual vacuum aspiration was offered to women. Onwards from 2003, medical abortion was offered up to 7 weeks gestation, and onwards from 2009, beyond 7 weeks too. Women who were eligible for both methods of abortion at the time they presented to the clinic, were offered a choice of MVA or medical abortion.

In the early years of service, all women opting for medical abortion were advised clinic use of misoprostol. After 2010, if the provider considered that a woman could be given home misoprostol based on distance of her home from the clinic, and access to a telephone or vehicle, then she was given a choice between home and clinic use of misoprostol.

A confidential form was used to collect information about women - including their socio demographic profile and prior use of contraception and abortion. For those who could not be provided abortion services at that clinic on the same day, the reasons were recorded. Contraceptive counseling was provided before abortion at the first visit. The clinics offered reversible contraceptives - oral pills, condoms, Depot_Medroxy Progesterone Acetate (DMPA) and Copper IUDs, while those opting for sterilization were subsequently referred to government or private facilities in Udaipur city.

The cost of abortion was fixed in recognition of the limited paying capacity of poor women of the area, and varied from Rs 200 to 500 during this period (approx. 4–9 USD), and varied with the procurement cost of medical abortion pills. This subsidized rate cost to the patient included the cost of consultation, procedures, supplies and medicines to be given before and after abortion, and the cost of bed charges in case woman remained in the clinic for use of misoprostol. ARTH offset the balance cost of providing these services from its programme grants and funds.

For those provided an abortion service, the type of abortion, post-abortion contraception and complications if any at the time of abortion were recorded. Between November 2013 and September 2014, additional information was gathered on the availability of a teelephone, personal transport facilities and whether any family members were aware about the abortion. Clinical information was recorded by nurses and doctors. After abortion, all women were provided instructions on danger signs, and were given a post abortion instruction sheet in the local language (Hindi).

All women were advised to return for a follow-up visit, and for those who did return, complications if any, were recorded. For those who did not return, a possible home follow-up visit was not carried out, for reasons of confidentiality. Onwards from 2013, the clinics were involved in two trial interventions to related to follow-up after medical abortion, hence data related to follow-up rates has been presented only up to 2012.

Strict attention was paid to confidentiality by all clinic staff. The doctors and nurses were periodically oriented on recent guidelines from Government of India and World Health Organization (WHO) regarding safe abortion, and were provided written guidance materials from time to time. Standard checklists were developed for assessment of eligibility, as was an information sheet containing post abortion instructions and guidance on home use of misoprostol.

### Statistical methods

Clinical case sheets of women with unwanted pregnancy were sent from the clinics to a central office once a month, where data was entered in Epi-Info 6 software. Data was analysed using Epi info and SPSS22 softwares. Descriptive analysis was conducted for most variables and Chi(2) test was used for statistical analysis.

## Results

Between August 2001 and March 2015, a total of 9076 women presented at the three clinics with unwanted pregnancy.

### Characteristics of women with unwanted pregnancies

Socio-demographic characteristics of women with unwanted pregnancies have been depicted in Table 1.

**Table 1:** Characteristics of women with unwanted pregnancies (*n* = 9076)

Residence		
• Rural	9076	100 %
Marital status		
• Married	8926	98 %
• Unmarried/widow/separated	150	2 %
Caste		
• Scheduled caste/tribe	5945	66 %
• Other	3131	34 %
Number of children		
• 0	481	5 %
• 1–2	3439	38 %
• 3–4	4022	44 %
• 5 or above	1134	12 %
Prior induced abortions		
• Yes	1821	20 %
• No	7255	80 %
Prior use of contraceptives		
• Yes	1294	14 %
Type of contraceptive used in the past		
• Oral pills	730	7.9 %
• Condom	548	5.9 %
• Copper-T	598	6.4 %
• DMPA	287	3.1 %
Gestational age in weeks (*n* = 8587 women who were pregnant)		
• Upto 7	2415	28.1 %
• 8–9	2871	32.7 %
• 10–12	2082	23.7 %
• 13 & above	1100	12.5 %
• Not recorded^a^	119	1.4 %
Person accompanying		
• No one	3007	33.1 %
• Husband	2886	31.8 %
• Family member other than husband	2266	25.0 %
• Health volunteer	515	5.7 %
• Neighbour/friend	402	4.4 %
Prior attempts of abortion		
• Any prior attempts of abortion (some women tried more than one method)	1129	12.4 %
○ Tablets from chemist shop	883	10.1 %
○ Decoction ^b^	229	2.6 %

^a^ Some women who arrived on a day when doctor was not available, did not undergo bimanual examination on the day of their first visit, and did not return to meet the doctor

^b^ Decoctions are usually made through boiling of some herbs

A majority of women were currently married, and nearly two thirds of women belonged to socio- economically underprivileged scheduled caste or tribe groups. Only 5 % were nulliparous, while 56 % had 3 or more children. One fifth had undergone a prior induced abortion, while less than a sixth had ever used a contraceptive.

About 12.5 % women came with pregnancies above 12 weeks. About a third of women came alone to the clinics, another third camewith their husbands, and the rest were accompanied by family members, friends or health volunteers.

**Fig. 1:**
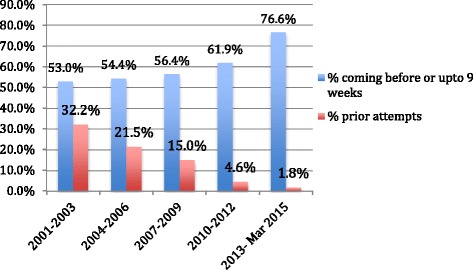
Prior attempts of abortion and gestational age

Almost 12.4 % of women had attempted to ‘bring on their period’ before coming to the clinic. On looking at the trend over the years, we found that in the early years of the service, nearly 33 % of women had tried out some remedy (usually over the counter ‘tablets’ or home made herbal decoctions) to abort the pregnancy, and over the years the proportion reduced to under 2 %. Over the same period, the proportion of women coming with earlier pregnancies increased (Fig. 1).

### Communication facilities at home and informants

Information on personal communication facilities and family awareness of the woman’s abortion is available for 1125 women that sought abortion after November 2013. Table 2 shows that 37 % had a personal (mobile) phone, another 45 % had access to their husband’s phone, while 16 % had no such access. In terms of personal transport facility, only a third had a two or four wheel vehicle at home, the rest had to rely on public transport. In almost all cases, husbands were aware that their wives were undergoing abortion, while other family members were in the know, only in a minority of instances.

**Table 2:** Availability of communication facilities and informants

	(*n* = 1235)
Availability of phone	
• Woman	456 (36.9 %)
• Husband	550 (44.5 %)
• Other	34 (2.8 %)
• None	195 (15.8 %)
Vehicle available at home	
• Four wheeler	19 (1.5 %)
• Two wheeler	397 (32.1 %)
• No vehicle/only bicycle	796 (64.5 %)
• NR	23 (1.9 %)
Who are aware that she is undergoing abortion	
• Husband	1175 (95.1 %)
• Anyone else other than husband	
○ Relatives from husband family	232 (18.8 %)
○ Relatives from natal family	83 (6.7 %)
○ Neighbour/other	66 (5.3 %)

### Provision of abortion service

**Fig. 2:**
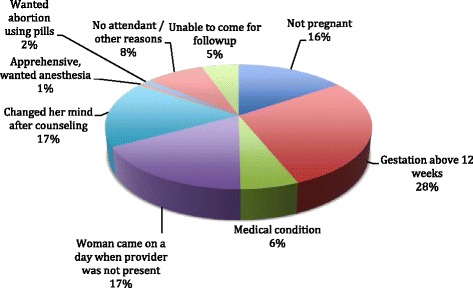
Reasons for not providing abortion service (*n* = 3284)

Of 9076 women presenting with unwanted pregnancy at the ARTH facilities, 6373 women (70 %) were provided abortion services. The reasons for not providing abortion service to 3284 women have been depicted in Fig. 2 - the most important reasons included gestation being above 12 weeks, woman coming to the clinic on a day when the doctor was not attending and change of mind after counseling. Since abortion services were available only on 1–2 fixed days per week when the doctor was visiting, women who came on other days were advised to return on the day of doctors’ visit. However, many of them did not return. A small fraction of women wanted abortion pills in the early years before medical abortion was approved in India. In the first year of service, providers being over-cautious, refused the service to those who had come alone. However, on realizing that that this precondition acted as a barrier for women who had often travelled for hours to reach the facility without an accompanying person, providers dropped this requirement. Furthermore, in the early years after starting the medical abortion service, (when evidence on home use of misoprostol and alternatives to clinic follow-up in low resource settings was unavailable), our service protocol mandated 3 clinic visits. Hence women who said they could not return to the facility for followup visit, were offered surgical evacuation, and if they refused this option, they were not offered an abortion service. As the service matured and more evidence on safe home use of misoprostol and (lack of) need for a follow-up visit became available, we changed our service delivery guidelines.

### Method of abortion

**Fig. 3:**
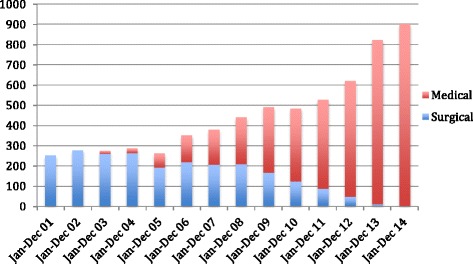
Type of abortion service provided at ARTH health centres (2001–2014, *n* = 6373)

In the first few years, only manual vacuum aspiration was offered to women. After 2003, women could be offered a choice of MVA or medical abortion, based on their gestational age. Over the years, the proportion of women opting for medical abortion increased (Fig. 3), such that in 2014, only 0.5 % women opted for MVA. Discussion with providers revealed that majority of women preferred medical abortion because of its non-invasive nature.

Because the gynecologist visited on specified days of the week and traveled back to the city late in the afternoon, ours was a periodic service. Hence MVA could only be provided to women who arrived in morning or by early afternoon, or about 2 h before closing time to allow for pre-abortion medication and analgesia to be given, and so that women could be observed at least for an hour before the doctor left the clinic. After medical abortion became available, it was possible to provide abortion services to women who arrived late, since the providers needed time only to counsel, screen for eligibility and to prescribe the pills.

Till 2012, all women receiving medical methods were asked to return for clinic use of misoprostol, but from 2013 onwards, providers began giving women the choice of home or clinic use of misoprostol. Among women who received medical abortion in 2013 and 2014, 38 % chose home use of misoprostol.

### Follow-up after abortion

Data related to follow-up rates within a month of initiating abortion is available for two clinics for the period 2001 to 2012 – it reveals that 52 % women returned for a clinic follow-up visit. The rate of women returning for follow-up was lower during 2001–03, when majority of abortions were surgical, and increased progressively along with increase in adoption of medical abortion (Table 3).

**Table 3:** Follow-up rates after abortion, 2001–2012 (*n* = 3868)^a^

	2001–2003 (*n* = 612)	2004–2006 (*n* = 897)	2007–2009 (*n* = 1265)	2010–2012^b^ (*n* = 1094)	Total (*n* = 3868)
Women who returned for follow-up	234	350	597	818	1999
% MTPs that were medical abortions	2.9 %	28.9 %	53.4 %	83.8 %	52.6 %
% follow-up	38.2 %	39.0 %	47.2 %	74.8 %	51.7 %

^a^ The data is presented for two clinics, where records of follow-up are maintained

^b^ The data is presented up to June 2012

### Post abortion contraceptives

**Fig. 4:**
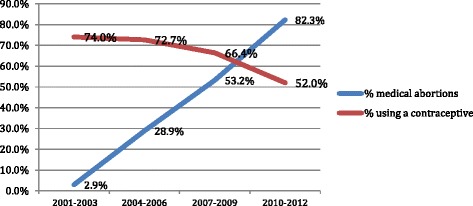
Monthly variations in total caseloads of ARTH clinics (2001-2012)

**Fig. 5:**
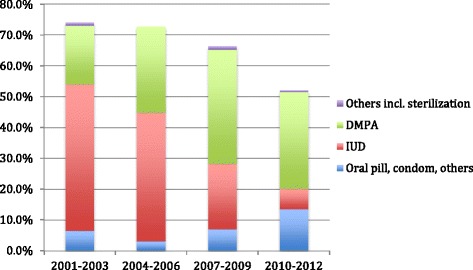
Proportion of women using a contracetive after abortion

Contraceptive use was recorded on the day MVA was provided or on day of mifepristone or clinic misoprostol. Subsequently if the woman came for follow-up visit, the use of contraceptives on the day of follow-up visit was recorded. However if a woman did not return for follow-up, then the information was available only up to the day of abortion. The data from 2001 to 2012 shows that the proportion of women using contraceptives after the abortion declined over the years from 74 to 52 % (Fig. 4). The method mix too, changed – while earlier on the intrauterine device (IUD) was the most common post-abortal method, over the years, the use of IUDs declined and that of combined oral pills, condoms and DMPA increased (Fig 5).

### Repeat abortion seekers

**Fig 6:**
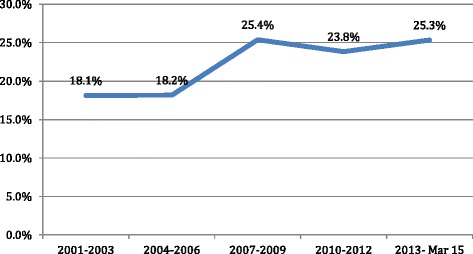
Trends in types of postabortion contraceptives adopted (2001–2012, *n* = 4247)

The proportion of women that had previously undergone abortion went up from 18 % in 2001–03 to 28 % in 2013–14 (Fig. 6). Women with three or more children were more likely to be seeking a second or subsequent abortion. Repeat users were also more likely to have used a contraceptive in the past (Table 4).

**Table 4:** Comparison of women coming for repeat abortion with those coming for the first time

	Repeat abortion seekers (*n* = 1821)	First time abortion seekers (*n* = 7255)
Number of children		
• 0–2 children	534 (29.3 %)	3386 (46.7 %)
• 3 or more children	1287 (70.7 %)	3869 (53.3 %)
Prior use of contraceptives		
• Yes	560 (30.8 %)	734 (10.1 %)
• No	1261 (69.2 %)	6521 (89.9 %)

### Monthly variations in abortion case load

**Fig 7:**
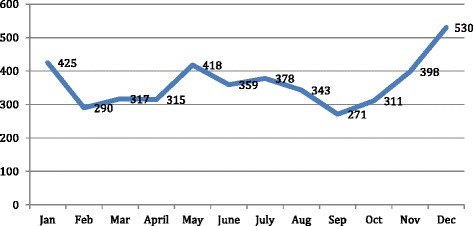
Proportion of abortion seekers who had repeat abortions

Data on average numbers of women with unwanted pregnancies over 12 years shows that, highest caseloads were seen in months of December, January and May. Lowest caseloads were seen in months of September, October and February (Fig. 7).

#### Representativeness of sample

No nationally representative data on profile of women with unwanted pregnancy is available, hence we were unable to compare women in our sample with a larger sample. Our study population was entirely rural, with 64 % belonging to the marginalized scheduled caste or tribe group, and in 2013–14, 38 % women owned a mobile phone. More recent data on education level among abortion seekers, available for 731 consecutive women in 2013–14, showed that 45 % women were literate [12]. In the state of Rajasthan, 75 % population was rural and 48 % women were literate [8]. In the districts in which ARTH’s clinics are located (Udaipur & Rajsamand), 46 % people belonged to underprivileged scheduled tribes or castes, and 30 % households owned a mobile phone in 2007-08 (expected to have increased by 2013–14) [13]. Hence we feel that, the profile of women seeking abortion at our facilities was similar to the population of the districts in which they are located, in terms of literacy levels and ownership of phones.

## Discussion

Despite a liberal law since 1972 and approval of medical abortion since 2003, the provision of legal or formal abortion services at primary care level in rural India continues to be limited. This analysis shows that provision of safe, legal abortion services at rural primary care facilities is feasible and can fulfill the needs of a large proportion of women with unwanted pregnancy. Our data also suggests that rural poor women, opt for safe abortion services as the first choice, if they are accessible, provided in a confidential and sensitive manner, and are affordable.

Our analysis shows that a large majority of women prefer medical over surgical abortion if given a choice, in line with other studies. For example, analysis of factors associated with abortion technique in France showed that given a choice, 84 % women adopted a medical method [14]. In other settings too, when women had information about both methods and were given a choice, they prefer medical abortion [15–17]. In our setting, medical abortion was not available till 2003, and subsequently was only offered to women till 7 weeks gestation, thereby rendering many ineligible. Later when the use of mifepristone-misoprostol was approved up to 9 weeks, and as provider experience grew, a greater proportion of women were considered eligible for medical abortion and were given a choice of methods. We believe that when choice is available, greater proportions of women choose medical abortion in order to avoid surgical intervention - fear of undergoing surgery is an important barrier in low-income countries [18].

Medical abortion provides an advantage when providers visit a facility for a limited number of hours. In our facilities, even if a woman arrived close to end of the working day when the provider was to leave, it was possible to provide medical abortion but not MVA, since the latter requires more preparation time.

The majority of women in our analysis were currently married, which is perhaps related to the low age at marriage in the area and state [9]. More than half the women had three or more children, indicating that abortion seekers are generally married women, who seek abortion to avoid yet another birth. From a community in which the socio-economically underprivileged scheduled caste or tribe groups constitute 47 % of the population, 66 % of women coming to our facilities belonged to this group – this is probably because our clinics provide highly subsidized, non-discriminatory services with differentially lower rates for the tribal community. During the period 2001 to 2015, the two rural blocks in which ARTH provided services did not have any other legal private abortion facility. Each block has one government Community Health Centre and 3–4 Primary Health Centres, which if staffed by a trained abortion provider, could have provided services. A review of annual reports of abortion service provision during two sample periods, 2007–10 (3 years) and during 2014–15 (1 year) revealed that none of these facilities reported performing a single abortion. While it is possible that a few abortions might have been performed without being reported, these were likely to be sporadically accessible in a clandestine manner. Hence, women seeking formal abortion service would either need to go to an urban clinic at one of the district headquarters (30 to 70 km away) or visit an ARTH facility. We therefore feel that women visiting ARTH facilities were representative of those seeking first trimester abortion in a rural community.

A pilot study from same rural area in 1998–99 showed that nearly 76 % of women with unwanted pregnancy who were referred to the city for an abortion, did not go to the city -- the majority continued with the pregnancy [11]. A few other studies from India shed light on profile of abortion seekers, however, all of them are from urban tertiary hospitals [19–21]. These studies too show that majority of the women seeking abortions were married women with one or more children.

Among women with unwanted pregnancy, less than a fourth had ever used a modern contraceptive. This to an extent is not surprising, given that information and utilization of reversible methods of contraceptives is limited in rural areas [9]. Government front-line workers seldom approach unmarried or recently married women regarding contraception, and in the bid to achieve programme objectives, the information they provide to women with two or more children is largely about sterilization. Our experience in the rural community suggests that there is a large pool of women that wishes to avoid sterilization despite not wanting more children, many abortion seekers are drawn from this pool.

After the abortion, 55 % women adopted a contraceptive on the day of abortion itself, or on day of follow-up visit. We provided counseling on all methods of contraception and women did not face any disincentives or pressure for not adopting contraceptives. A recent study from Nepal has shown similar rates of initiation of contraception after medical or surgical abortion [22]. The preference of type of contraceptive changed over the years. Initially, the copper IUD was the most common method, but its use declined over the years. The popularity of DMPA increased over the years, yet the overall initiation of contraception declined over the years. We believe that this was related to increase in the proportion of medical abortion. A study from eastern India has shown that the women who underwent medical abortion were significantly less likely to adopt contraception in the month following abortion compared to those undergoing surgical abortion (58 % vs 86 %), although the difference narrowed by the end of the second month [23]. A study from Australia has also shown that immediate provision of long acting reversible contraceptives (LARC) was more likely after surgical than after medical abortion [24].

The proportion of women who had attempted to ‘bring on their periods’ before coming to the ARTH facility reduced over the years. The reason for this, we believe, is that as information about the availability of our abortion service increased over the years, women started coming directly to our clinics. Secondly, there is a strong preference for avoiding a surgical procedure. In the early years when medical abortion was not available, several women who wanted to avoid surgical evacuation had tried out (mostly ineffective) non-invasive methods such as herbal tablets or decoctions, and came to the clinic after these methods failed. In later years, when availability and information about (mifepristone-misoprostol) medical abortion increased, more women started coming directly to the facility. A study from India has shown that 31 % women had made at least one unsuccessful attempt to terminate the pregnancy [25].

Our results indicate 52 % came for a follow-up visit after abortion, although the proportion increased over the years. Because of the large fraction of women that did not follow up during 2001–2012, it was difficult to accurately report the rates of complications for the entire period. However, we have data available from a research trial nested within these facilities during 2013–14 [12]. In this study, follow-up contact was established with 97.5 % and the rate of complications (defined as hemorrhage requiring blood transfusion or IV fluids, infection requiring IV antibiotics, or hospitalization) was 0.3 %. About 4 % women had incomplete abortion and 1 % had ongoing pregnancies. Given that service protocols did not change for the trial, we feel this result would hold true, for the entire period.

Over the years, the proportion of women who came for repeat abortions increased, however, despite the increase, the majority were first time users. Compared to first time users, repeat users were more likely to have had three or more children, and also having used a contraceptive in the past. Women who had received an abortion service in the past had likely been counseled and provided a contraceptive, while first time users did not have the opportunity, hence repeat users were more likely to have used a contraceptive in the past. In contrast to our findings, data from countries, such as France and Estonia, shows that repeat abortion seekers are more likely to be young women or students living alone [26, 27]. Our results indicate that women who wish to limit their families face difficulties in meeting their contraceptive needs, possibly because of limited access to long-acting reversible contraceptives (LARCs). India’s national programme lays heavy emphasis on female sterilization, which however, often suffers from poor service quality [28]. In order to enable more women to fulfill their reproductive health needs, a greater choice of LARCs needs to be available at affordable cost through public health system. This is also likely to reduce the need for abortion among women who have completed their families.

In our study, among rural women, 37 % had a phone, and only 34 % had a vehicle at home. This finding has two implications – first, providers need to consider that it might not be easy for rural women to easily reach a health facility for want of transport, hence they should try to reduce the unnecessary visits. For example, women should be provided abortion at the first visit, should be given a choice to use misoprostol at home [29] and should be offered alternatives to routine follow-up [12]. Second, although several programmes have started using phones to transmit messages related to the abortion process [30], mobile phones have still not reached the majority of rural women in India. Hence women in low resource settings would need to be given detailed verbal instructions along with context specific written material at the first visit itself. For women owning a phone, reminders or further instructions could be provided on phone.

Our data also showed that most of the time, only the husband was aware that his wife was undergoing an abortion, other persons were aware in less than a third of cases. This further highlights the need for service providers to maintain confidentiality. Further, in case a woman needs support (e.g., visiting a clinic in the event of a side effect or help with housework), they would need to draw on support from husbands.

Data on monthly variations shows that greater number of women coming during months of December-January and in May. Seasonal variations in delivery rates, induced abortion rates and conception rates have been reported earlier in different settings [31, 32]. In United States, for example, higher caseloads for induced abortions were reported from January through April. We have not systematically explored reasons for higher conception rates during the months preceding December – January and May. Relatively lower conception rates around August-September could possibly be linked to the custom of abstinence among tribal persons during one specific month around this time. Service delivery sites in India should be prepared to deal with higher caseloads during certain months.

### Strengths and limitations

The strength of this study is that it documents results from a primary care setting in an interior rural area of India, while most of other studies on abortion service provision are from urban areas or from tertiary level facilities. Our area represents underserved areas of the country, where mortality and morbidity related to unsafe abortion is likely to be higher due to restricted access to the service. Further, our study presents data over 14 years, and its results provide information on changing patterns in profile of women, method of abortion provided and chosen, changes in contraception and followup rates. This understanding can be used to inform policy and planning service delivery. The limitation of this analysis is that we do not have complete data for some of the variables for the entire period. This occurred because this was a service intervention that kept evolving with time.

## Conclusions

To conclude, our experience shows that medical abortion allows expansion of access to primary care settings. Health facilities in rural primary care settings may not be able to provide surgical abortion, because of lack of providers skilled to perform surgical evacuation or equipment. It would be feasible to provide medical abortion in such settings. Our experience also shows that when offered a choice of method, women exhibit strong preference for medical abortion, hence medical abortion should be offered as an option to all women seeking abortion. To improve access, it is also important to remove other barriers related to abortion services such as those related to accompanying person, insistence on use of specific contraceptive methods, confidentiality, privacy and cost [3, 7, 33, 34].
