# Erratum to: Improving access to safe abortion in a rural primary care setting in India: experience of a service delivery intervention

**DOI:** 10.1186/s12978-016-0196-y

**Published:** 2016-07-20

**Authors:** Kirti Iyengar, Sharad D. Iyengar

**Affiliations:** Action Research & Training for Health, Satyam, Ramgiri, Badgaon, Udaipur, Rajasthan 313011 India; Department of Women’s & Children’s Health, Karolinska Institutet, Solna, Stockholm 17176 Sweden

## Erratum

After publication of the original article [1], it came to the authors’ attention that an instruction submitted at proofing had been misinterpreted by the Production team. Figures 4–7 were reordered, but the original Figure captions were unfortunately retained, meaning that these Figures’ captions were all incorrect.

The captions for Figs. 4–7 have now been corrected in the original article.
